# Supplementary material for: Genomic Alterations Associated with Estrogen Receptor Pathway Activity in Metastatic Breast Cancer Have a Differential Impact on Downstream ER Signaling
Source: Cancers (Basel). 2023 Sep 4;15(17):4416. doi: 10.3390/cancers15174416 (PMC10487136; doi:10.3390/cancers15174416)
Supplement: Supplementary file 1 [file cancers-15-04416-s001.zip › cancers-2559693-supplementary.pdf]

Supplementary Figures

A.

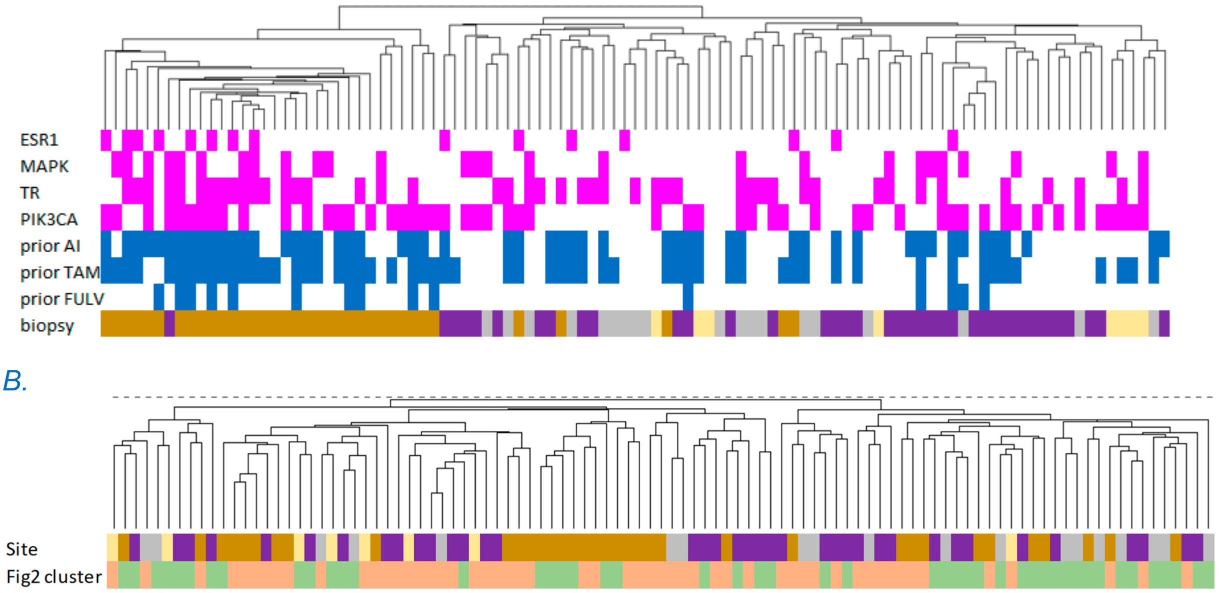

Supplementary Figure S1. (A) When applying unsupervised clustering on the top 5000 variable genes, the RNA sequencing data revealed two large clusters of tumors that segregated primarily by the biopsy site. The first cluster of samples mainly consisted of biopsies obtained from liver metastases (brown) and the second cluster included samples from various organ sites consisting on bone (yellow), lymph node (grey), and other biopsy sites (purple). Gene expression in the first cluster was mainly driven by gene expression of normal liver tissue. We therefore corrected for this bias by performing an ComBat correction. **(B)** After correction for this bias, we then re-clustered the samples, showing a much more diverse pattern in the biopsy sites and that the sample groups identified by clustering ESR1 target genes (i.e. the clusters from Figure 2, green represent samples in cluster A; orange represent samples in cluster B) are distributed over the unsupervised clusters.

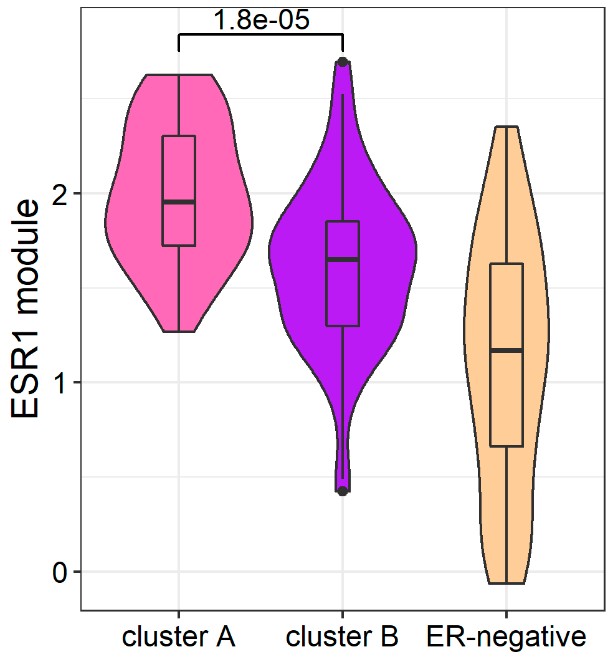

Supplementary Figure S2. Validation of the ER target genes used, by evaluating the *ESR1* module (35), a gene signature associated with an active ER pathway. This module provides weights and which ESR module specific genes to use, with the module score calculated as the average (expression x weight) of the ESR module specific genes. Here we show that the samples from cluster A have a significant higher (p=1.8e-5, Mann-Whitney) module score than the samples in cluster B, while the module score of cluster B was in between those from cluster A and ER-negatives.

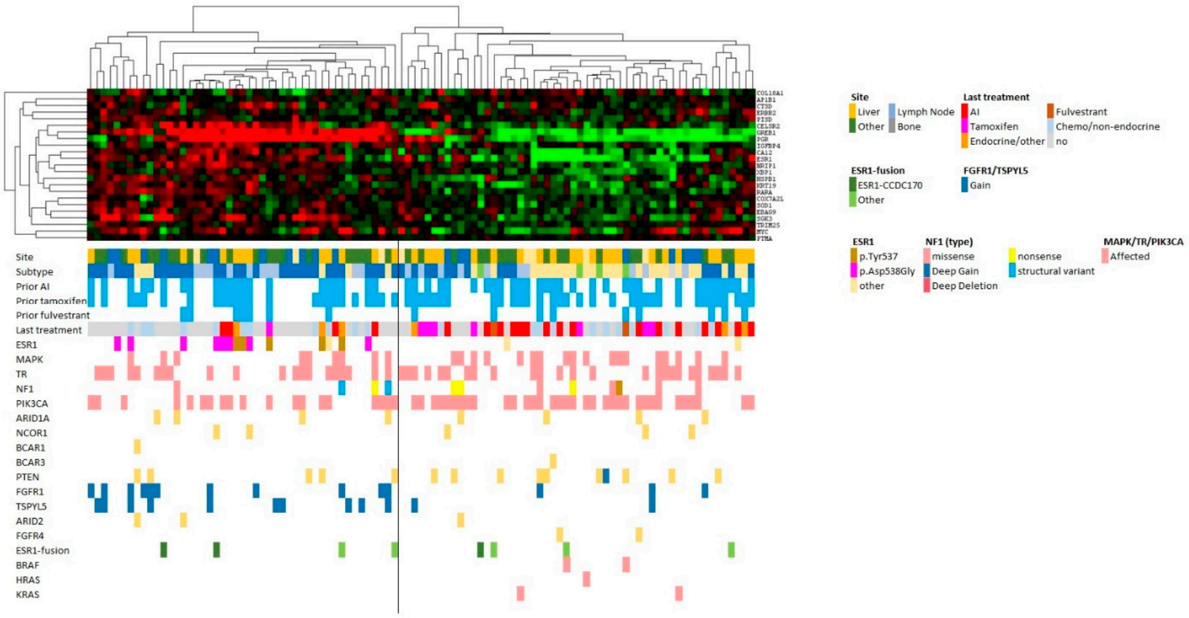

Supplementary Figure S3. Hierarchical clustering based on ER-target genes (see methods for the list of genes on which this clustering was based) also including genes which were not significantly different between both clusters. Mutations in *ESR1* p.Tyr537 indicate either the mutation p.Tyr537Asn or p.Tyr537Ser.
